# Positive Relational Management and Occupational Well-Being: The Mediating Role of Flourishing and Organizational Citizenship Behaviors

**DOI:** 10.3390/ejihpe14120199

**Published:** 2024-12-10

**Authors:** Marta Peña, Marta Llorente-Alonso, Cristina Garcia-Ael, Gabriela Topa

**Affiliations:** 1 Faculty of Health Sciences, European University Miguel de Cervantes, 47012 Valladolid, Spain; mpena@uemc.es; 2 Faculty of Health Sciences of Soria, University of Valladolid, 42004 Soria, Spain; 3 Faculty of Psychology, National University for Distance Education (UNED), 28040 Madrid, Spain

**Keywords:** positive relational management, flourishing, organizational citizenship behaviors, life satisfaction

## Abstract

This study examines the relevance of interpersonal relationships in the work environment, focusing specifically on analyzing associations between positive relational management, which refers to the use of relational resources that enable adaptation to the workplace, and key organizational variables such as flourishing, individual-directed organizational citizenship behaviors (OCBis), and life satisfaction. Given the importance of this topic, a structural model is required for the possible relationship between positive relational management and other organizational variables relevant to occupational well-being. As a preliminary step, the Positive Relational Management Scale (PRMS) was analyzed and validated in a sample of 348 Spanish workers. The results revealed that the overall model has a good fit, with reliable and valid construct measures. Moreover, the three-dimensional structure of the model was confirmed, although gender invariance was not satisfied. In conclusion, the results confirm the simple mediation hypothesis, in which flourishing mediates the relationship between positive relational management and life satisfaction. In contrast, multiple mediations between the variables could not be confirmed. This study highlights the importance of interpersonal relationships for employee well-being in the workplace.

## 1. Introduction

The work environment constitutes a vital area in which psychosocial challenges can have a significant impact on employee well-being and burnout [1]. Positive psychology offers a promising approach to addressing these challenges by focusing not only on difficulties but also on enhancing people’s strengths in all areas of their social life, including the workplace. Organizations seek a positive approach that allows them to benefit from the positive characteristics of their employees [2]. In particular, healthy organizations promote the use of employees’ strengths [3].

From this perspective, it is crucial to examine the variables that foster healthy organizations, such as, for example, interpersonal relationships. These relationships are a key factor in most aspects of life and are especially important in organizations since they form part of what is known as “social capital”, or, in other words, the resources obtained from social networks, engagement, and reciprocity between individuals [4], and play a key role in protecting health [5]. Fostering this social capital is important, as it helps individuals achieve personal and professional goals [6]. Several studies have shown that positive interpersonal relationships at work promote pleasure and happiness [7,8,9].

To date, studies on interpersonal relationships at work have focused mainly on examining the nature, content, and quality of these relationships [10] or have sought to explore civic relationships in the workplace [11], and little attention has been paid to the importance of managing these relationships in a positive manner. To address this gap, di Fabio and Kenny [12] developed the Positive Self and Relationship Management (PS and RM) Model, in which positive relationship management is a key element.

In light of the above, the general aim of the present study is to analyze the relationship between positive relational management and other individual variables that are relevant to work-related well-being, such as flourishing, organizational citizenship behaviors (OCBs), and life satisfaction.

### 1.1. Interpersonal Relationships and the PS and RM Model

The Positive Self and Relational Management (PS and RM) Model emphasizes both the individual and the relational strengths that enable individuals to face the challenges posed by their daily lives. Specifically, it seeks to cultivate these strengths by promoting the management of oneself and one’s relationships, adopting a preventive perspective and fostering well-being [12]. The model has been validated using different variables, including (among others) positive and negative affect, authenticity, emotional intelligence, and reflection on one’s life project [12]

The PS and RM model comprises three constructs: (a) Positive Lifelong Life Management, which refers to well-being and satisfaction with life and includes aspects such as authenticity; (b) Positive Lifelong Self-Management, which is based on the resources managed at an individual level and includes aspects such as professional adaptation skills, self-concept, and self-efficacy; and (c) Positive Lifelong Relational Management, which refers to the relational resources that allow people to adapt in the workplace and includes aspects such as social skills, emotional intelligence, and social support [12]. To measure positive relational management, the model proposes the Positive Relational Management Scale (PRMS), the psychometric properties of which were reported by Di Fabio [13] using the variables Perceived Social Support and Life Meaning, among others. Other studies have used the sustainability of human capital [14] and other variables that seek to promote well-being [15].

### 1.2. Interpersonal Relationships, Life Satisfaction, and Flourishing

Several studies have found that interpersonal relationships at work have a decisive influence on different organizational variables. Two variables that have been linked to positive relational management are life satisfaction [11,12,13] and flourishing [11,13]. Flourishing is defined as the achievement of a balanced life in which people feel good [16]. Findings point to a significant and positive correlation between positive relational management and life satisfaction [12].

Well-being is made up of two aspects, one hedonic and the other eudaimonic [17]. Hedonic well-being has an affective and a cognitive component and is related to subjective well-being [18], social support, and emotional intelligence [19]. Life satisfaction would be included here. In contrast, research into eudaimonic well-being focuses more on optimal functioning [17], including flourishing.

Research has shown that higher levels of job satisfaction and social support at work are linked to greater emotional intelligence and more flourishing [19]. Moreover, flourishing correlates significantly with positive affect [20], as work teams with high flourishing levels are characterized by positive communication and expressions of support among team members [21].Life satisfaction and flourishing in the workplace therefore promote positive outcomes in terms of both individual and organizational well-being [22]. Work relationships play a key role in well-being. In the study by Colbert et al. [23], the authors conclude that work relationships support employee flourishing, benefiting both workers and organizations. Research across different populations has demonstrated important links between social connections, well-being, and life satisfaction. A study of Turkish students found that social connectedness significantly predicts well-being [24]. Similarly, researchers have established a strong positive relationship between life satisfaction and flourishing [25]. Furthermore, Younes and Alzahrani [26] found that flourishing mediates the relationship between life satisfaction and mindfulness. Based on the mediating role of life satisfaction and previous evidence of a relationship between the proposed variables, the following assumptions were made:

**Hypothesis** **1.** *Positive relational management is positively and significantly associated with life satisfaction.*

**Hypothesis** **2.** *The relationship between positive relational management and life satisfaction is mediated by flourishing in a simple mediation process.*

### 1.3. Interpersonal Relationships, OCBs, Life Satisfaction, and Flourishing

OCBs include all employee activities that go beyond the formal requirements of the job and make a major contribution to the effectiveness of organizational functioning. Well-being is an important antecedent of this prosocial activity [27]. One proposal for how the variability of these behaviors is organized suggests the existence of two dimensions: the first (individual-directed citizenship behavior, OCBi) involves prosocial behaviors directed at individuals within the organization (e.g., altruism and courtesy) and places more emphasis on well-being at work; and the second (organization-directed citizenship behavior, OCBo) encompasses behaviors directed at the organization as a whole (e.g., civic virtue and conscientiousness) [28].

In a study focused on OCBs and flourishing, Okikechukwu et al. [29] found that, in a group of nurses, flourishing played a positive predictive role in OCBs (the higher their flourishing levels, the more the individuals in question engaged in OCBs). Similarly, flourishing was also found to be positively associated with OCBs in a sample of secondary school teachers [30].

Individual OCBs have been found to be related to life satisfaction, mediated by positive affect [31]. They also have a positive influence on interpersonal relationships, except when they are selfish, in which case, their effect on the quality of the relationships is negative [32].

Based on the association found between OCBs, flourishing, life satisfaction, and interpersonal relationships, the following hypothesis is proposed:

**Hypothesis** **3.** *The relationship between positive relational management and life satisfaction will be mediated by flourishing and OCBs in a multiple mediation process.*

Finally, before examining the relationship between positive relational management and the proposed relational variables flourishing, OCBs, and life satisfaction, and prior to testing the structural model, we validated the Spanish adaptation of the “Positive Relational Management Scale”. The “Positive Relational Management Scale” (PRMS), which measures respect, care, and relationships, is viewed as opening up new avenues of research and intervention in the business context from the perspective of positive prevention [13]. We also analyzed gender differences in relation to this variable since gender invariance has already been proven in relation to life satisfaction [33], flourishing [34], and OCBs, albeit with certain nuances [35]. In light of the above, the following hypothesis is put forward:

**Hypothesis** **4.** *Gender invariance will be confirmed for the structural Positive Relationship Management Model.*

## 2. Materials and Methods

### 2.1. Participants

The present study was carried out with 348 Spanish workers (58.62% women) from different organizations, aged between 18 and 70 years (M = 40.73; SD = 11.72). Participants lived in different Autonomous Communities in Spain (See Table 1). In terms of qualifications, 60.64% claimed to have completed higher education, 18.97% had vocational training, 10.34% had a bachelor’s degree, 6.04% had primary level qualifications, 3.45% had a master’s degree, and 0.57% had a PhD. As for the professional sector in which they worked, 73.85% were employed in the service sector and 26.15% in the industrial sector. Mean organizational seniority was 11.08 years (SD = 11.43). 

### 2.2. Instruments

To fulfill the study aims, participants completed the scales outlined below.

The Positive Relational Management Scale (PRMS) [13] comprises 12 items rated on a Likert-type scale ranging from 1 (Strongly Disagree) to 5 (Strongly Agree). The scale is composed of three four-item subscales: (a) respect (example item: “I respect the value and uniqueness of others”); (b) caring (example item: “I usually take care of others”); and (c) connectedness (example item: “I maintain good relationships with my family”). In this study, a Cronbach’s alpha of 0.87 was obtained for the overall scale. The values obtained for each subscale (α respect = 0.78; α caring = 0.64; α connectedness = 0.81) were very similar to those obtained by Di Fabio [13] (α overall = 0.85; α respect = 0.82; α caring = 0.80; α connectedness = 0.81), with the exception of the caring subdimension.

To measure flourishing, we used the Spanish adaption by Pozo et al. [36] of the Flourishing Scale developed by Diener et al. [37]. The scale comprises eight items that measure the respondent’s flourishing (e.g., “I lead a useful and meaningful life”) on a response scale ranging from 1 (Strongly disagree) to 5 (Strongly agree). The Cronbach’s alpha was 0.88, very similar to the value obtained in both the original scale (α = 0.87) and the study by Pozo et al. [36] (α = 0.88 in the Colombian sample and 0.85 in the Spanish sample).

Organizational citizenship behaviors were measured using the Spanish adaptation by Dávila and Finkelstein [38] of Lee and Allen’s Organizational Citizenship Behavior Scale [39]. The scale comprises 16 items divided into two dimensions: 8 items for OCBis and 8 items for OCBos. Only the OCBis dimension was used in this study. Items are rated on a 5-point Likert-type scale ranging from 1 (Never) to 5 (Always). An example of an item would be “I selflessly spend my time helping others who have work-related problems”. The Cronbach’s alpha for OCBis was 0.83 [40].

To measure life satisfaction, we used the Spanish adaptation [41] of Diener et al.’s abbreviated Satisfaction With Life Scale. This brief 5-item scale measures quality of life (e.g., “The conditions of my life are excellent”) using a response scale ranging from 1 (Strongly disagree) to 5 (Strongly agree). The Cronbach’s alpha obtained was 0.88. Finally, participants also provided sociodemographic data (age, education level, etc.).

### 2.3. Procedure

Questionnaires were completed over a six-month period, and participants were recruited through incidental sampling, using a snowball sampling technique. The questionnaire was developed and distributed through various different channels in order to ensure that it reached a broad, varied group of individuals, with the primary criterion being that all respondents had to be in active employment. Data were collected online. Participants, who were all volunteers, first completed the informed consent form and then the questionnaire. Anonymity and the confidentiality of the answers were guaranteed. This study followed a cross-sectional design.

### 2.4. Statistical Analysis

First, descriptive statistics and bivariate correlations of the study variables were calculated using the IBM SPSS Statistics 26 statistical program. Specifically, a variance-based structural equation model (SEM) was developed using the partial least squares (PLS) method [42]. The data were analyzed using the SmartPLS statistical software (v.4) [42].

In the present study, the decision to use PLS-SEM was based on the following considerations: (a) the procedure is recommended by Barroso et al. (p. 429) [43]., as it allows researchers to simultaneously assess the reliability and validity of the theoretical construct measures (measurement model) and estimate the relationships between constructs (structural model); (b) the model approach involves higher-order modeling, simultaneously modeling lower-order and higher-order constructs. Since we planned to use latent variable scores in subsequent analyses, the PLS-SEM is the recommended option (p.48) [44]; (c) the construction of a hierarchical component model implies that some constructs will be measured reflectively and others formatively; and (d) PLS is able to handle small sample sizes and is exempt from the assumption of normality; it is therefore recommended for social science research [45]. Furthermore, “when using large amounts of data (N ≥ 250), CB-SEM and PLS-SEM results tend to be quite similar, as long as there are a number of indicators (4 or more) to measure each of the constructs” (p. 53) [44].

In this study, we adopted a novel approach based on forward modeling, using a hierarchical component model. These higher-order models allow for a reduction in the number of relationships in the structural model, resulting in a simpler PLS nomogram (p. 66) [44]. Specifically, the guidelines recommended by Sarstedt et al. [46] in the two-step disjoint approach were followed. Moreover, positive relational management was conceptualized as a reflective–reflective construct type. We started with the two-step decoupled approach (the disjoint two-stage approach) [46], including the lower-order components (respect, caring, and connectedness) of the higher-order construct (positive relational management) in the nomogram. The lower dimensions were linked by pathways to the other constructs to which the higher-order construct is related (flourishing and life satisfaction). Next, the PLS algorithm was run with the lower-order constructs estimated in A mode.

After running the model and checking that it was well modeled, the latent variable scores were saved, and a new data file containing these data was created. This enabled the second step in the approach to be completed. The scores extracted from the latent variables in the previous step were used to model the higher-order construct. The rest of the nomogram constructs were evaluated using their indicators, in the same way as in the first step.

## 3. Results

Table 2 shows the means, standard deviations, and correlations pertaining to the study variables.

### 3.1. First Stage of the Two-Step Decoupled Approach

First, the model assessment focused on the reflective measurement models of the lower-order components, which must satisfy all relevant criteria (internal consistency, convergence, validity, and discriminant validity) [46]. See Table 3 and Table 4.

The initial assessment of the lower-order constructs (respect, care, and connectedness) revealed adequate composite reliability values. Only the caring dimension failed to obtain high rho_A and reliability (Cronbach’s α) values. The average extracted variance value was close to 0.50. We also calculated discriminant validity using the HTMT criterion, finding validity among the variables in our study. Only the dimensions caring and respect obtained values of above 0.85.

**Figure 1:**
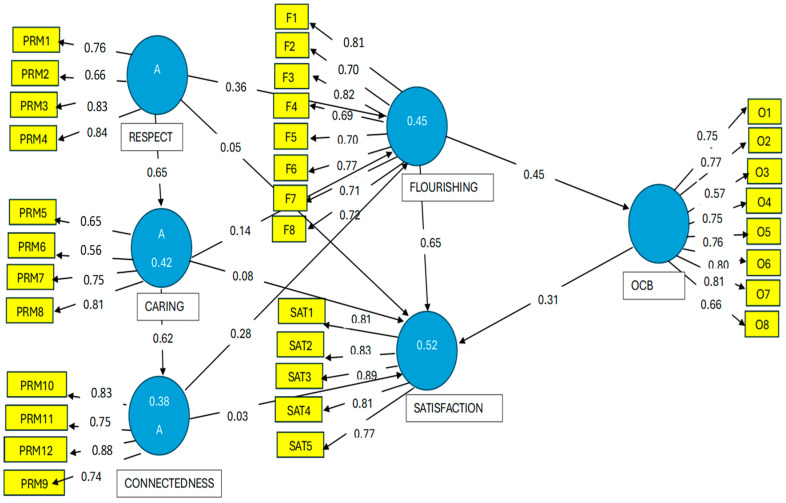
Stage 1 of the dissociated two-step approach [46]. *Note*: Execution of the PLS algorithm. We present the factor loadings or simple correlations between each indicator and its construct; standardized paths or β coefficients between constructs; coefficients of determination (R^2^) = value within constructs. OCB: organizational citizenship behaviors. PRM = positive relational management; O = organizational citizenship behavior; F = flourishing; SAT = satisfaction).

Next, we measured the simple loadings or correlations of the indicators with their corresponding construct. Correlations with a value of over 0.707 [47] indicate adequate fit. Furthermore, according to Hair et al. [48], when assessing the simple loadings of the indicators, those with values of between 0.40 and 0.70 should be removed from the scale if this leads to an increase in composite reliability. As shown in Figure 1, in the caring and respect dimensions, there were two items with values below 0.707. We therefore eliminated those items and ran the PLS algorithm again, with the results indicating no improvement in the composite reliability. Specifically, when item 6 was removed, the composite value dropped to 0.80, and when item 2 was also eliminated, it continued to be lower than the initial value with all the items included. The decision was therefore made to keep these items (see Figure 1).

Secondly, after having checked the correct modeling of the first step, we saved the scores of the latent variable (scores) and included them in a new data file. Then, we continued with the second step of the approach, in which the scores extracted from the latent variables in the previous step were used to model the higher-order construct. The rest of the nomogram constructs were evaluated using their indicators, in the same way as in the first step. In this second stage, the higher-order construct positive relational management was made up of the lower-order constructs connectedness, caring, and respect, as indicators, and assessed in a reflective model based on the common factor model.

We then interpreted the PLS model, which comprises three phases: (a) global model assessment, (b) a measurement model (external model), and (c) a structural model (internal model).

### 3.2. Global Model

Using the Standardized Root Mean Square Residual (SRMR) criterion, we obtained a value of 0.067 in the saturated model and 0.07 in the estimated model, thereby indicating an adequate fit of the global model. The SRMR measures the difference between the observed correlation matrix and the correlation matrix implied by the model. Hu and Bentler (p. 27) [49] proposed SRMR values of <0.08 as being indicative of good data fit.

### 3.3. Measurement Model

**Figure 2:**
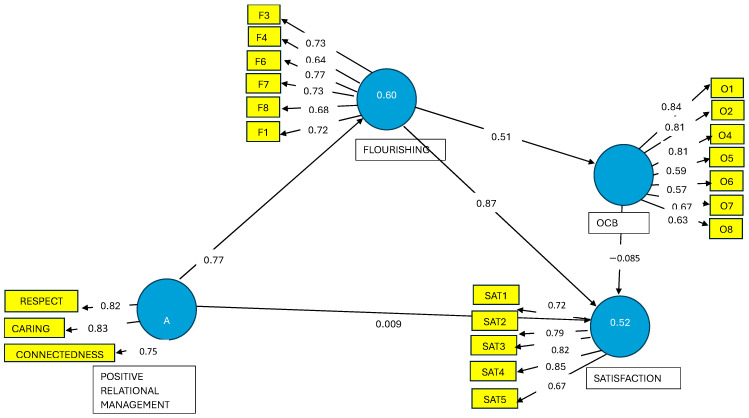
Stage 2 of the dissociated two-step approach [46]. *Note*: Execution of the Consistent PLS algorithm. We present factor loadings or simple correlations between each indicator and its construct; standardized paths or β coefficients between constructs; coefficients of determination (R^2^) = value within constructs. O, OCB = organizational citizenship behaviors, F = flourishing, SAT = satisfaction.

To assess the measurement model, we first ran the consistent PLS algorithm and analyzed the factor loadings of the model. We eliminated items 2 and 5 of the flourishing variable, with values of 0.60 and 0.50, respectively, and item 3 of the organizational citizenship behaviors variable, with a value of 0.30. We observed that the average extracted variance rose from 0.48 to 0.50 in the case of flourishing and from 0.48 to 0.51 in the case of organizational citizenship behaviors. We also analyzed the increase in composite reliability, which rose from 0.87 to 0.88 in the case of organizational citizenship behaviors (see Figure 2).

Next, we assessed the reflective measurement model following the process outlined by Hair et al. [44], which includes composite reliability to assess internal consistency, and individual indicator reliability and average variance extracted (AVE) to assess convergent validity. We also assessed discriminant validity. See Table 5.

We also calculated the heterotrait–monotrait (HTMT) ratio. Henseler et al. [50]. showed that the HTMT ratio is better able to detect a lack of discriminant validity than other methods such as cross-loadings and the Fornell–Larcker criterion. In a model with adequate fit, heterotrait correlations should be smaller than monotrait correlations, meaning that the HTMT ratio should be below 1. According to Kline [51]., the HTMT ratio should be below 0.85, and Gold et al. [52] established an even less restrictive value of 0.90. In this study, all the variables had lower values, enabling us to confirm discriminant validity (see Table 6).

### 3.4. Structural Model

Having verified that the constructs were both reliable and valid, we then assessed the structural model. First, we assessed the collinearity of the structural model using the variance inflation factor (VIF). This value must be less than or equal to 5 [48]. As shown in the table below, all VIF values were below 5, indicating the absence of collinearity between the predictors (see Table 7).

**Figure 3:**
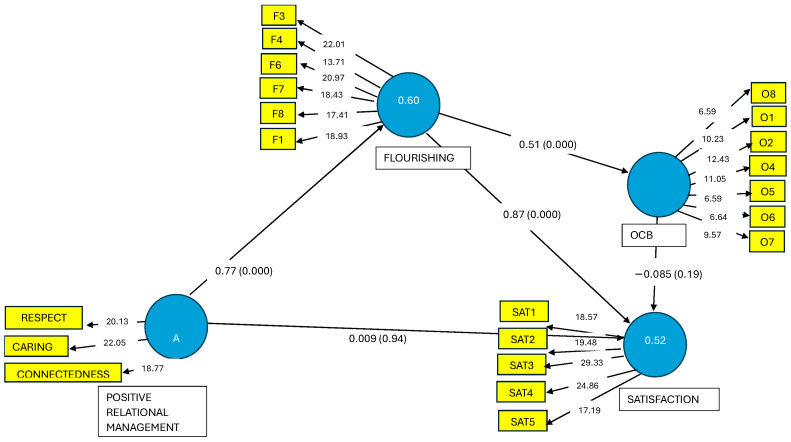
Stage 2 of the decoupled two-step approach [46]. Note: consistent bootstrapping run. We present t values between indicators and their construct; coefficients of determination (R^2^) = value within constructs; path coefficients and significance levels between constructs. F = flourishing; O, OCB = organizational citizenship behavior, SAT = satisfaction.

Subsequently, we evaluated the algebraic sign, magnitude, and statistical significance of the path coefficients (see Figure 3). The signs of the path coefficients coincided with the initially postulated hypotheses. The highest standardized β coefficient values were found between the variables positive relational management and flourishing (β = 0.77, *p* < 0.000) and between flourishing and satisfaction with life (β = 0.86, *p* < 0.000).

The significance of the path coefficients was assessed using bootstrapping for consistent PLS (5000 subsamples). The association between flourishing and organizational citizenship behaviors was found to be significant (β = 0.50, *p* < 0.000), whereas the relationships between positive relational management and life satisfaction (β = 0.009, *p* = 0.93) and between organizational citizenship behaviors and life satisfaction (β = −0.08, *p* = 0.19) were not.

Table 8 and Table 9 present the total and indirect effects of the variables in the present study.

Regarding the coefficient of determination, the model explained 60% of the variance observed for flourishing, 26% of the variance observed for organizational citizenship behaviors, and 69.8% of the variance observed for life satisfaction. These results support Hypotheses 1 and 2, although not Hypothesis 3.

### 3.5. MICOM Model: Analysis of Measurement Model Invariance

In order to determine whether or not the group differences in the model estimates were due to the different content or meaning of the latent variables between the groups, the MICOM model (p.345) [44] was calculated.

Henseler et al. [50] developed a three-step procedure for calculating the measurement invariance of composite models (MICOM). The procedure is appropriate in our model because variance-based SEM techniques model latent variables as composites [49]. 

#### 3.5.1. Step 1: Configuration Invariance

We analyzed whether or not a composite was specified equally across all groups [50] by performing an initial qualitative assessment that ensured that the same indicators were used in each measurement model, and the data were treated identically.

#### 3.5.2. Step 2: Composite Invariance

To evaluate composite invariance, we performed a permutation algorithm with PLS (5000 permutations), in which the selected groups were, on the one hand, men, and on the other hand, women. To test for compound invariance, the original correlation must be greater than or equal to the 5% quantile. The MICOM results presented in Table 4 indicate that the composite scores did not differ between the two groups (see Table 10).

#### 3.5.3. Step 3: Assessment of the Equality of Means and Variances of the Composite Variables

We assessed whether the original differences in means and variances were between 2.5% and 97.5%. If so, then this would indicate complete invariance. If one of these variances were to fall between 2.5% and 97.5%, this would indicate partial invariance of means and variances (see Table 11).

After having confirmed partial measurement invariance, since the values for organizational citizenship behaviors were significant, we then moved on to the multigroup analysis [44]. The results revealed differences between the two gender groups in relation to the caring and flourishing dimensions (β = −0.02, *p* < 0.02), as well as the life satisfaction and flourishing dimensions (β = 0.85, *p* < 0.002). Specifically, the association between caring and flourishing was only significant among women (see Table 11). The indirect effects that were only significant among women were between respect–caring–flourishing (β = 0.14, *p* < 0.002), respect–caring–flourishing–life satisfaction (β = 0.07, *p* < 0.006), respect–caring–flourishing–individual-directed organizational citizenship behaviors (β = 0.06, *p* < 0.01), caring–flourishing–individual-directed organizational citizenship behaviors (β = 0.10, *p* < 0.005), and caring–flourishing–life satisfaction (β = 0.12, *p* < 0.004). (See Table 12).

## 4. Discussion

The main aim of the present study was to examine the relationship between positive relational management and key variables for occupational well-being, such as flourishing, individual-directed organizational citizenship behaviors (OCBis), and life satisfaction.

Prior to analyzing the structural relationships, we analyzed the Positive Relational Management Scale, finding that the overall model had a good fit, with adequate reliability and validity values for all the construct measures. These results indicate that positive relational management is not a unidimensional concept but is made up of three dimensions: respect, caring, and connectedness. These findings confirm the validation of the scale in the Spanish context, consistent with that reported by previous studies conducted with Italian employees [13] and New Zealand managers [7]. Furthermore, the structural equation modeling process corroborated the three-dimensional structure posited in previous research [13]. 

When addressing psychosocial challenges and well-being in the workplace, it is crucial to understand positive relationship management and its impact on key aspects such as flourishing, organizational citizenship behaviors, and employee life satisfaction.

Our results confirm Hypotheses 1 and 2. Positive relationship management is significantly and positively associated with life satisfaction (Hypothesis 1), and flourishing mediates the relationship between positive relationship management and life satisfaction, thereby confirming the existence of a simple mediation (Hypothesis 2). These results are consistent with those found in previous studies [11,12,13] and highlight the importance of promoting and developing flourishing, given its mediating role. Positive organizational psychology strives to emphasize workers’ potential, and confirming the associations that exist between these three variables will enable Human Resources departments to implement interventions that benefit employees, not only at a personal level but at a group level also, by including relational and organizational variables, as well as others that impact organizational health, such as life satisfaction and flourishing. 

Hypothesis 3, in contrast, was not confirmed. OCBis and flourishing were not found to be mediating variables in a composite mediation between positive relationship management and life satisfaction. Unlike those reported in previous studies, our data do not indicate a relationship between OCBs and flourishing [29,30] or between OCBs and life satisfaction [31]. Consequently, although these variables are known to be associated with each other, the hypothesis tested in this study, i.e., that they are linked through the individual dimension of OCBs as part of a composite mediation process, was not confirmed. 

The most important results observed pertain to gender differences, a finding that fails to confirm Hypothesis 4. The structural model was only replicated in women, not in men. This reflects the stereotypes that are entrenched in our culture regarding women being responsible for taking care of those around them. The female stereotype is usually related to aspects such as emotionality, sensitivity, and tenderness, which, together with motherhood, means that, historically, women have been attributed the role of domestic and personal carers. Our results, which indicate better performance among women in care-related fields, confirm the stereotype of women in the workplace, in both high- and low-status positions, being oriented more towards relationships, respect [53,54], and care, associated with a strong domestic tradition [55].

In conclusion, the measurement of positive relational management proposed by the PS and RM model is of vital importance due to the benefits of relational resources for women. As reported previously in the literature, interpersonal relationships play a key role in people’s well-being [7]. Consequently, good interpersonal relationships facilitate work [8], influence well-being [7]., and are associated with happiness [9] and OCB [28], and as we have indeed been able to demonstrate, this construct not only has a three-dimensional structure but is also subject to simple mediation relationships involving flourishing, life satisfaction, and OCB, with flourishing playing a mediating role in the associations between these variables.

### Strengths, Limitations, and Future Research

The main strength of this study is that it confirms the three-dimensional concept of positive relational management. Moreover, prior to this, an instrument already validated in other countries such as Italy [13] and New Zealand [56] was validated in a Spanish sample. The findings presented here attest to the importance of having instruments that evaluate relational resources at work since these resources form part of the social capital that, together with social mechanisms and organizational practices, helps develop positive labor capital [6]. Studying these aspects not only improves the work environment but also enhances mental health. This is because, among other aspects, social capital is associated with mental health due to the fact that the social networks that form it promote social cohesion, provide protection in times of crisis, impact public health, and are associated with economic development and an improvement in work outcomes [57].

Interpersonal relationships at work are therefore a key part of work success, and studying their management is of vital importance. Relationally enriched workplaces can foster psychological states that, with respect to the beneficiaries of the work itself, boost motivation [58]. Also, leadership for human capital sustainability has been found to be a positive relationship resource in organizations that links to individual eudaimonic well-being [59]. Li et al. [60] conducted a longitudinal study on nurses in China, observing a positive association between relationship-oriented human resource management and employee motivation. On the other hand, a study with teachers by Nethavhani and Percy [61] found that management support improved the well-being programs implemented in the company. For all the reasons outlined above, it is important to continue conducting research that adds to the existing body of theory and evidence and confirms these results in different countries. Moreover, the use of concepts from positive psychology, such as life satisfaction and flourishing, which promote the achievement of healthier organizations, is encouraged. Given the relationships that exist between all these variables, it would be interesting to continue to study their associations, either with simple mediations, which were confirmed in this study, or with multiple relationships, which in this case were not confirmed.

Despite this, however, this study has certain limitations that need to be taken into consideration and improved upon in future research. The first is the reliability of the caring subscale. Although Di Fabio [13] obtained a Cronbach’s alpha of 0.82, in the present study, the value obtained was 0.65, with a Rho_A of 0.67, which is low. Also, despite having improved the number of participants in the sample with respect to the Italian sample recruited by Di Fabio [13] (348 Spaniards compared to 251 Italians), the scale needs to be analyzed further in increasingly larger samples. It would also be interesting to validate it in other countries and to translate it into different languages. As in the study carried out in New Zealand [56], which focused on managers, it would be interesting to sample not workers in general, as we did in the present study, but to focus specifically on certain work areas (health, law enforcement, etc.). Finally, it would also be interesting to carry out longitudinal studies using this scale.

## 5. Conclusions

This study contributes to the growing body of research on positive organizational psychology by examining the relationships between positive relational management (PRM) and key aspects of occupational well-being. Our findings confirm the three-dimensional structure of PRM, consisting of respect, caring, and connectedness, validating its applicability in the Spanish context.

The results reveal a significant positive relationship between PRM and life satisfaction, with flourishing mediating this relationship. This underscores the importance of fostering flourishing in the workplace as a pathway to enhanced life satisfaction. However, contrary to our expectations, individual-directed organizational citizenship behaviors (OCBis) did not mediate the association between PRM and life satisfaction nor did we find support for a composite mediation model involving both flourishing and OCBis.

Interestingly, our study revealed notable gender differences, with the structural model holding true only for women. This finding highlights the persistence of gender stereotypes in the workplace, particularly regarding caregiving roles and relational orientations.

### Practical Implications

These results have important implications for organizational practice, emphasizing the need for Human Resources departments to develop interventions that cultivate positive relationships and promote flourishing among employees. Such initiatives can benefit both individual well-being and organizational health.

While this study provides valuable insights, it also has limitations, including the relatively low reliability of the caring subscale and the need for larger, more diverse samples. Future research should aim to validate the PRM scale in different cultural contexts and specific occupational sectors. Additionally, longitudinal studies would be beneficial to examine the long-term effects of PRM on occupational well-being.

In conclusion, this study underscores the significance of positive relational management in the workplace, particularly in terms of its impact on flourishing and life satisfaction. By fostering positive relationships and promoting flourishing, organizations can enhance employee well-being and potentially improve overall organizational outcomes.

## Figures and Tables

**Table 1:** Demographics data.

	*N*	%	*M*	*SD*
Age	348		40.73	11.72
Gender				
Female	204	58.62		
Male	144	41.38		
Autonomous Communities				
Madrid	82	23.56		
Castilla y Leon	79	22.7		
Galicia	62	18.39		
Catalonia	13	3.74		
Extremadura	7	2.01		
Basque Country	6	1.72		
Valencian Community	4	1.15		
Balearic Islands	4	1.15		
Andalusia	2	0.57		
Murcia	2	0.57		
Castilla La Mancha	2	0.57		
Ceuta	1	0.29		
Not Indicate	84	23.56		
Seniority			11.08	11.43
Education Level				
Higher Education	211	60.64		
Vocational Training	66	18.97		
Bachelor’s Degree	36	10.34		
Primary Level	21	6.04		
Master’s Degree	12	3.45		
PhD	2	0.57		
Professional Sector				
Service Sector	257	73.85		
Industrial Sector	91	26.15		

**Table 2:** Bivariate correlations, means, and standard deviations.

Variables	*M*	*SD*	1	2	3	4	5	6
Respect	4.18	0.54	-					
Caring	3.87	0.62	0.63 **	-				
Connectedness	4.18	0.68	0.54 **	0.60 **	-			
*Positive Relational Management*	4.08	0.52	0.83 **	0.88 **	0.86 **	-		
*Flourishing*	4.08	0.60	0.59 **	0.52 **	0.65 **	0.65 **	-	
*Life Satisfaction*	3.57	0.77	0.47 **	0.45 **	0.53 **	0.70 **	0.70 **	-
*OCBis*	3.88	0.67	0.49 **	0.43 **	0.49 **	0.44 *	0.31 **	0.31 **

*Note: N* = 348. * *p* < 0.05, ** *p* < 0.01. Higher-order constructs are in italics. OCBis = individual-directed organizational citizenship behaviors.

**Table 3:** Reliability and validity statistics.

	A	Rho_A	Composite Reliability ρC	AVE	Discriminant Validity
Respect	0.78	0.78	0.86	0.61	0.78
Caring	0.65	0.67	0.79	0.49	0.70
Connectedness	0.82	0.84	0.88	0.65	0.80
*Life satisfaction*	0.88	0.89	0.91	0.68	0.82
*Flourishing*	0.88	0.89	0.91	0.55	0.74
*OCBis*	0.88	0.89	0.90	0.54	0.73

*Note:* Rho_A = Dijkstra–Henseler’s rho (ρA); A = Cronbach’s alpha; AVE = average variance extracted; higher-order constructs are in italics. OCBis = individual-directed organizational citizenship behaviors. Discriminant validity is the square root of the variance extracted (AVE).

**Table 4:** Discriminant validity assessed using the HTMT criterion.

	Caring	*Flourishing*	*OCB*	Connectedness	Respect	*Life Satisfaction*
Caring						
*Flourishing*	0.70					
*OCBis*	0.62	0.50				
Connectedness	0.83	0.65	0.45			
Respect	0.90	0.72	0.61	0.68		
*Life Satisfaction*	0.59	0.79	0.36	0.52	0.57	

*Note:* Higher-order constructs are in italics. OCBis = individual-directed organizational citizenship behaviors.

**Table 5:** Measurement model: construct reliability, convergent validity, and discriminant validity.

	A	Rho_A	CR	AVE	Discriminant Validity
*Flourishing*	0.86	0.86	0.86	0.50	0.70
*Positive Relational Management*	0.82	0.82	0.82	0.60	0.77
*OCBis*	0.88	0.88	0.88	0.51	0.71
*Life Satisfaction*	0.88	0.88	0.88	0.60	0.77

*Note:* CR = Composite reliability. Rho_A = Dijkstra–Henseler’s rho (ρA). AVE = Average variance extracted. A = Cronbach’s alpha. Higher-order constructs are in italics. OCBis = Individual-directed Organizational Citizenship Behaviors. Discriminant validity represents the square root of the variance extracted (AVE).

**Table 6:** Discriminant validity evaluated using the HTMT criterion.

	*Flourishing*	*Positive Relational Management*	*OCB*	*Life Satisfaction*
*Flourishing*				
*Positive Relational Management*	0.77			
*OCB*	0.50	0.64		
*Life Satisfaction*	0.82	0.62	0.35	

*Note:* Higher-order constructs are in italics. OCB = organizational citizenship behaviors.

**Table 7:** Variance inflation factor.

Variables	VIF
Caring	2.08
Connectedness	1.73
Respect	1.84
OCB1	1.89
OCB2	1.89
OCB4	1.91
OCB5	2.22
OCB6	2.58
OCB7	2.37
OCB8	1.60
Sfs1	2.50
Sfs3	2.49
Sfs4	1.62
Sfs6	1.83
Sfs7	1.81
Sfs8	1.57
Satisfaction1	2.13
Satisfaction2	2.51
Satisfaction3	3.28
Satisfaction4	1.88
Satisfaction5	1.92

*Note:* flourishing (Sfs); organizational citizenship behaviors (OCB); Satisfaction = life satisfaction; VIF = variance inflation factor.

**Table 8:** Total effects.

	β	SD	*t*	*p*
Flourishing -> OCBis	0.51	0.05	9.35	0.00
Flourishing -> Life Satisfaction	0.82	0.09	8.83	0.00
Positive Relational Management -> Flourishing	0.77	0.04	17.25	0.00
Positive Relational Management -> OCBs	0.39	0.05	6.95	0.00
Positive Relational Management -> Life Satisfaction	0.64	0.05	11.76	0.00
OCBis -> Life Satisfaction	−0.08	0.06	1.29	0.19

*Note:* OCBs = organizational citizenship behaviors.

**Table 9:** Indirect effects.

	β	*SD*	*t*	*p*
Positive Relational Management -> Flourishing -> OCBis	0.39	0.05	6.95	0.00
Positive Relational Management -> Flourishing -> Life Satisfaction	0.67	0.09	7.33	0.00
Flourishing -> OCBis -> Life Satisfaction	−0.04	0.03	1.21	0.22
Positive Relational Management -> Flourishing -> OCBis -> Life Satisfaction	−0.03	0.02	1.18	0.23

*Note:* OCBis = individual-directed organizational citizenship behaviors.

**Table 10:** MICOM. Stage 2 results.

	Original Correlation	Correlation of Permutation Means	5.0%	*p*-Values of the Permutation
Caring	0.99	0.99	0.98	0.92
Flourishing	0.99	0.99	0.99	0.31
OCBis	0.99	0.99	0.99	0.49
Connectedness	1	0.99	0.99	0.81
Respect	0.99	0.99	0.99	0.55
Life satisfaction	1	0.99	0.99	0.88

*Note*: OCBis = individual-directed organizational citizenship behaviors.

**Table 11:** MICOM. Stage 3 results.

	Mean—Original Differences (Mean—Differences of Permutation Means)	2.50%	97.50%	*p*-Values of Permutation	Variance—Original Difference (Variance—Difference of Permutation Means)	2.50%	97.50%	*p*-Values of Permutation
Caring	−0.19	−0.21	0.21	0.08	0.25	−0.28	0.28	0.08
	−0.002				(−0.002)			
Flourishing	−0.09	−0.21	0.21	0.41	0.3	−0.38	0.37	0.13
	−0.001				0			
OCBis	−0.274	−0.2	0.21	0.009	0.3	−0.26	0.27	0.02
	−0.001				0			
Connectedness	−0.176	−0.2	0.21	0.09	0.16	−0.33	0.32	0.32
	−0.002				(−0.003)			
Respect	−0.077	−0.21	0.2	0.48	0.24	−0.26	0.25	0.06
	−0.001				0			
Life satisfaction	−0.064	−0.21	0.21	0.55	0.15	−0.29	0.3	0.3
	0				0			

OCBis = individual-directed organizational citizenship behaviors.

**Table 12:** Multigroup analysis of direct effects (men and women).

	Path Coefficients Men	Path Coefficients Women	*p*-Values Men	*p*-Values Women
Caring -> Flourishing	−0.01	0.23	0.81	0.001
Caring -> Connectedness	0.66	0.57	0	0
Caring -> *Life Satisfaction*	0.02	0.13	0.72	0.10
Flourishing -> OCBis	0.49	0.43	0	0
Flourishing -> *Life Satisfaction*	0.85	0.51	0	0
*OCBis* -> *Life Satisfaction*	−0.11	0.005	0.07	0.94
Connectedness -> Flourishing	0.36	0.21	0	0.001
Connectedness -> *Life Satisfaction*	−0.10	0.09	0.19	0.18
Respect -> Caring	0.70	0.60	0	0
Respect -> Flourishing	0.47	0.28	0	0.001
Respect -> Life Satisfaction	0.05	0.02	0.48	0.78

## Data Availability

The data presented in this study are only available upon request from the corresponding authors due to participants’ privacy issues.
